# 1634. Respiratory Syncytial Virus-Associated Health Care Utilization in the Pivotal Phase 3 Trial RSV Vaccine Efficacy Study In Older Adults Immunized Against RSV Disease (RENOIR)

**DOI:** 10.1093/ofid/ofad500.1468

**Published:** 2023-11-27

**Authors:** Edward E Walsh, Kumar Ilangovan, Agnieszka Zareba, Qin Jiang, Gonzalo Pérez Marc, Tjitte Verbeek, Johannes Breedt, Helen Stacey, Akiyoshi Uchiyama, Jussi Ojanperä, Elliot N DeHaan, Michael Patton, Yanqing Kan, Daniel P Eiras, Tarek Mikati, Elena Kalinina, David Cooper, Annaliesa S Anderson, Kena A Swanson, William C Gruber, Alejandra C Gurtman, Beate Schmoele-Thoma

**Affiliations:** University of Rochester, Rochester, NY; Pfizer, Vaccine Research and Development, Raleigh, North Carolina; Pfizer, Pearl RIver, New York; Pfizer, Pearl RIver, New York; Hospital Militar Central de Buenos Aires, Ciudad Autónoma de Buenos Aires, Buenos Aires, Argentina; Huisartsenpraktijk Radesingel, Radesingel, Groningen, Netherlands; Emmed Research, Pretoria, Gauteng, South Africa; Diablo Clinical Research, Walnut Creek, California; Medical Corporation Asbo, Tokyo Asbo Clinic, Kyobashi Chuo-ku, Tokyo, Japan; Terveystalo Jyväskylä, Jyväskyla, Keski-Suomi, Finland; Pfizer, Pearl RIver, New York; Pfizer, Vaccine Research and Development, Raleigh, North Carolina; Pfizer, Pearl RIver, New York; Pfizer, Inc., Pearl River, New York; 3. Pfizer, Inc., Vaccine Research & Development, Pearl River, New York; Pfizer, Pearl RIver, New York; Pfizer, Pearl RIver, New York; Pfizer, Pearl RIver, New York; Pfizer, Pearl RIver, New York; Pfizer, Pearl RIver, New York; Pfizer, Pearl RIver, New York; Pfizer, Pearl RIver, New York

## Abstract

**Background:**

Respiratory Syncytial Virus (RSV)-related disease poses an economic burden due to a substantial amount of health care utilization (HCU). RENOIR is a phase 3 global, multicenter, randomized, double-blinded, placebo-controlled study evaluating vaccine efficacy (VE) in adults ≥60 years of age during two RSV seasons in Northern and Southern Hemisphere countries (Argentina, Canada, Finland, Japan, Netherlands, South Africa, and USA) (NCT05035212). VE at the end of the first RSV surveillance season (EOS1) against ARI-RSV, Lower Respiratory Tract Illness (LRTI)-RSV with ≥2 symptoms (2+ LRTI-RSV), and LRTI-RSV with ≥3 symptoms (3+ LRTI-RSV) was 62.2% (44.4, 74.9), 65.1% (35.9, 82.0), and 88.9% (53.6, 98.7), respectively.

**Methods:**

HCU data and concomitant corticosteroid or antibiotic use were collected during the RSV season for Acute Respiratory Illness (ARI) events (defined as more than 1 day of new or increased cough, nasal congestion/discharge, sore throat, wheezing, sputum production, or shortness of breath). A pre-planned analysis of HCU at EOS1 was performed to assess HCU among participants receiving RSVpreF vs. placebo.

**Results:**

A higher proportion of RSV-associated HCU was associated with more severe symptoms (43.0%, 60.3%, and 75% for ARI-RSV, 2+ LRTI-RSV, and 3+ LRTI-RSV, respectively). Medically attended (MA) VE was similar to VE for all first-episode cases with MA ARI-RSV 65.1% (35.9, 82.0), MA 2+ LRTI-RSV 70.4% (33.0, 88.4), MA 3+ LRTI-RSV 84.6% (32.0, 98.3). Most RSV-associated HCU were outpatient visits, with more in the placebo arm. A higher proportion of placebo arm participants with RSV cases had an RSV-associated emergency room visit. For ARI-RSV, there were 3 hospitalizations in the placebo arm and none in the RSVpreF arm. The placebo arm had higher antibiotic (11.8 - 55.6% higher) or corticosteroid (2.8 – 44.4% higher) use compared to the RSVpreF arm for ARI- or LRTI-RSV events.

Table 1

**Figure:**
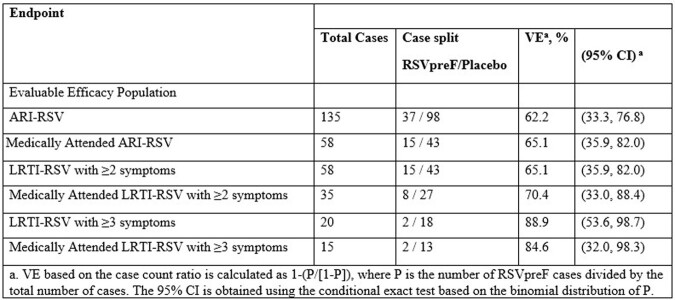

Vaccine Efficacy of RSVpreF Against First Episode of RSV Cases in the First RSV Season – Evaluable Efficacy Population

Table 2

**Figure:**
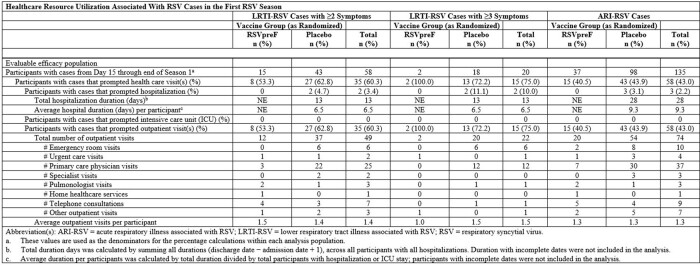

Healthcare Resource Utilization Associated With RSV Cases in the First RSV Season

Table 3

**Figure:**
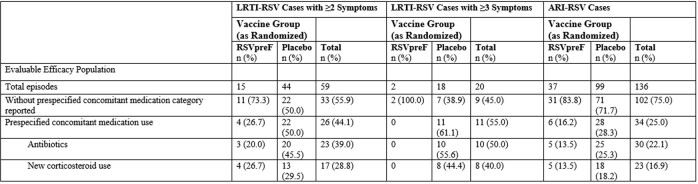

Prespecified Category of Concomitant Medication Uses for RSV Cases in the First RSV Season

**Conclusion:**

RSVpreF reduced overall HCU and antibiotic or corticosteroid treatment for RSV-associated illnesses. The highest VE among MA RSV-associated cases was for MA 3+ LRTI-RSV. These findings suggest that RSVpreF may reduce RSV-related healthcare needs in older adults, thus alleviating this burden on health systems.

**Disclosures:**

**Edward E. Walsh, MD**, Icosavax: Advisor/Consultant|Merck: Advisor/Consultant|Merck: Grant/Research Support|Merck: Honoraria|Moderna: Advisor/Consultant|Pfizer: Grant/Research Support **Kumar Ilangovan, MD, MSPH, MMCi**, Pfizer, Inc.: Employee|Pfizer, Inc.: Stocks/Bonds **Agnieszka Zareba, MD PhD**, Pfizer: Employee|Pfizer: Stocks/Bonds|Pfizer: Stocks/Bonds **Qin Jiang, PhD**, Pfizer: Employee|Pfizer: Employee|Pfizer: Stocks/Bonds|Pfizer: Stocks/Bonds **Gonzalo Pérez Marc, M.D.**, GSK: Grant/Research Support|Merck: Grant/Research Support|Moderna: Expert Testimony|Moderna: Grant/Research Support|Pfizer: Grant/Research Support **Elliot N. DeHaan, MD**, Pfizer: Employee|Pfizer: Stocks/Bonds **Michael Patton, B.Sc.**, Pfizer Inc.: Employee|Pfizer Inc.: Stocks/Bonds **Yanqing Kan, MS**, Pfizer: Pfizer's employee|Pfizer: Stocks/Bonds **Daniel P. Eiras, MD, MPH**, Pfizer, Inc.: Stocks/Bonds **Tarek Mikati, MD,MPH**, Pfizer: Stocks/Bonds **Elena Kalinina, PhD**, Pfizer: Pfizer employee|Pfizer: Stocks/Bonds **David Cooper, PhD**, Pfizer, Inc.: Stocks/Bonds **Annaliesa S. Anderson, PhD**, Pfizer: Employee|Pfizer: Stocks/Bonds **Kena A. Swanson, Ph.D.**, Pfizer: Employee|Pfizer: Stocks/Bonds **William C. Gruber, MD**, Pfizer, Inc.: Employee|Pfizer, Inc.: Stocks/Bonds **Alejandra C. Gurtman, M.D.**, Pfizer: Employee|Pfizer: Stocks/Bonds **Beate Schmoele-Thoma, MD**, Pfizer: Stocks/Bonds

